# A New 3D Iodoargentate Hybrid: Structure, Optical/Photoelectric Performance and Theoretical Research

**DOI:** 10.3390/molecules28248033

**Published:** 2023-12-10

**Authors:** Jun Li, Shuyue Xie, Ming Pang, Jiacheng Zhu, Jinting Wu, Yongdi Zhang, Bo Zhang

**Affiliations:** 1 College of Chemistry and Chemical Engineering, Shandong Provincial Key Laboratory and Collaborative Innovation Center of Chemical Energy Storage & Novel Cell Technology, Liaocheng University, Liaocheng 252059, China; shuyuexie_lcu@163.com (S.X.); mpang_lcu@163.com (M.P.); jczhu_icu@163.com (J.Z.); jtwu_lcu@163.com (J.W.); ydzhang_lcu@163.com (Y.Z.); 2 State Key Laboratory of Structural Chemistry, Fujian Institute of Research on the Structure of Matter, Chinese Academy of Sciences, Fuzhou 350002, China

**Keywords:** iodoargentate, 3D microporous material, optical behavior, photocurrent response, theoretical research

## Abstract

The explorations of new three-dimensional (3D) microporous metal halides, especially the iodoargentate-based hybrids, and understanding of their structure-activity relationships are still quite essential but full of great challenges. Herein, with the aromatic 4,4′-dpa (4,4′-dpa = 4,4′-dipyridylamine) ligands as the structural directing agents, we solvothermal synthesized and structurally characterized a novel member of microporous iodoargentate family, namely [H_2_-4,4′-dpa]Ag_6_I_8_ (**1**). Compound **1** possesses a unique and complicated 3D [Ag_6_I_8_]*_n_*^2*n*−^ anionic architecture that was built up from the unusual hexameric [Ag_6_I_13_] secondary building units (SBUs). Research on optical properties indicated that compound **1** exhibited semiconductor behavior, with an optical band gap of 2.50 eV. Under the alternate irradiation of light, prominent photoelectric switching abilities could be achieved by compound [H_2_-4,4′-dpa]Ag_6_I_8_, whose photocurrent densities (0.37 μA·cm^−2^ for visible light and 1.23 μA·cm^−2^ for full-spectrum) compared well with or exceeded those of some high-performance halide counterparts. Further theoretical calculations revealed that the relatively dispersed conduction bands (CBs) structures in compound **1** induced higher electron mobilities, which may be responsible for its good photoelectricity. Presented in this work also comprised the analyses of Hirshfeld surface, powder X-ray diffractometer (PXRD), thermogravimetric measurement, energy-dispersive X-ray spectrum (EDX) along with X-ray photoelectron spectroscopy (XPS).

## 1. Introduction

Iodoargentate-based hybrids, combining the advantages of both organic and inorganic components, continue to captivate researchers by virtue of their rich structural chemistry and distinctive photophysical properties [1,2]. Among them, 3D microporous architecture characteristics of regular holes or channels are of special importance and have drawn increasing attention in recent years, mainly benefiting from their immense breakthrough in the domains of semiconductor, photocatalysis, adsorption, nonlinear optics, white-light emission, photochromism/thermochromism and piezoelectricity/ferroelectricity [3,4,5,6,7,8,9].

Structurally, the iodoargentate family possesses the myriad SBUs that were built up from the condensations of flexible primary building units (PBUs) ([AgI*_x_*]; *x* = 2, 3, 4, 6) via vertex/edge/face-sharing and the short Ag*···*Ag contact. Some representative examples include [Ag_5_I_6_], [Ag_3_I_7_], [Ag_5_I_12_], [Ag_2_I_5_], [Ag_6_I_11_], [Ag_4_I_8_], [Ag_7_I_13_], [Ag_5_I_9_], [Ag_10_I_18_] and [Ag_6_I_12_] [1,2]. Up to now, a large number of iodoargentate hybrids based on the above-mentioned SBUs have been solvothermal harvested and structurally characterized, whose anionic moieties customarily involve in some low-dimensional motifs (e.g., discrete polynuclear clusters, infinite chains/ribbons or expanded layers) [10,11,12,13,14,15,16,17]. Comparatively speaking, the 3D microporous compounds have rarely been documented which may be largely attributed to the weak host-guest interactions and poor template-directing effects. Among the numerous templates, the multi-pyridine derivatives, especially those with suitable sizes/shapes/configurations, stand out due to their good structural modification abilities, having been widely exploited in the construction of new microporous metal halides. Especially, many studies have shown that some of them behave optically active and their embedding may endow the as-obtained products with certain desired functionalities [5,6,18,19]. This progress has been chiefly witnessed by some photochromic, thermochromic and photocatalytic materials, as exemplified by [*N*-Bz-Py]_4_Ag_9_I_13_ (*N*-Bz-Py^+^ = *N*-benzylpyridinium), [(Me)_2_-2,2′-bipy]Ag_8_I_10_ ((Me)_2_-2,2′-bipy = 1,1′-dimethyl-2,2′-bipyridinium), [DMBTz]_2_Ag_5_I_7_ (DMBTz^+^ = dimethylbenzotriazolium) and [MCMP]Ag_3_I_4_ (MCMP^+^ = 1-methyl-4-(carbomethoxy)pyridinium) [5,6,18,19]. Noticeably, the photoelectrochemical research of non-perovskite microporous iodoargentates is still in its infancy. Therefore, the design and development of a new number of 3D microporous iodoargentates and understanding their photoelectrochemical behaviors remain urgently needed but challenging.

On the basis of the above consideration, we undertook systematic studies for exploring new 3D microporous derivatives under the solvothermal condition. Fortunately, by employing the aromatic 4,4′-dpa ligands as the structural modifiers, we successfully constructed a novel and complicated hybrid iodoargentate, namely [H_2_-4,4′-dpa]Ag_6_I_8_ (**1**). The optical study showed that compound **1** has an optical band gap of 2.50 eV, implying the nature of semiconductor properties. More attractively, compound [H_2_-4,4′-dpa]Ag_6_I_8_ exhibited good photoelectric conversion abilities, with the visible light and full-spectrum on/off current density of 0.37 μA·cm^−2^ and 1.23 μA·cm^−2^, respectively. Particularly, such photocurrent densities were comparable with or largely outperformed those of some high-performance halide counterparts that may be ascribed to the stronger mobility of photo-induced electrons. Given here also consists of the analyses of the Hirshfeld surface, thermogravimetric test, PXRD, EDX and XPS.

## 2. Results

### 2.1. Structural Description of Compound **1**

**Figure 1:**
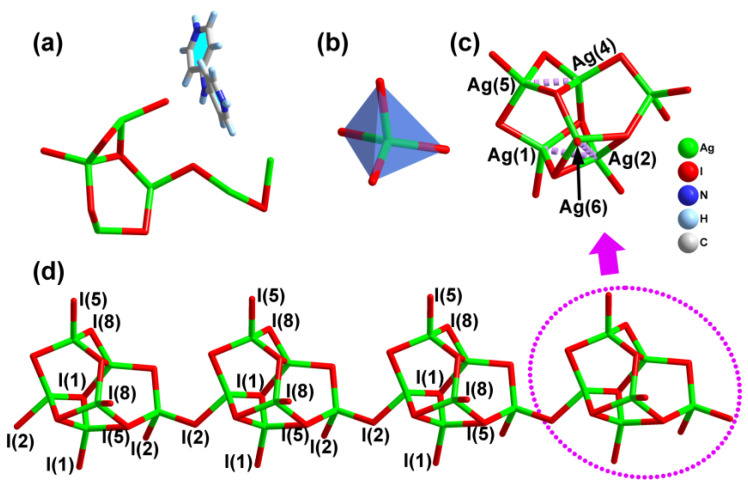
(**a**) The asymmetric unit of compound **1**. (**b**) The tetrahedral [AgI_4_] building block. (**c**) The [Ag_6_I_13_] moiety shows Ag···Ag interaction. (**d**) The chain-like [Ag_6_I_12_]*_n_*^6*n*−^ anion, in which the dotted circle and arrow were used to indicate the [Ag_6_I_13_] moiety.

Compound **1** belongs to the monoclinic crystal system (P2_1_/n space group, No. 14), as disclosed by single crystal X-ray diffraction analyses. It features a unique 3D [Ag_6_I_8_]*_n_*^2*n*−^ anionic framework, involving the 1D channels fulfilled by the template cations (Figure 1). Its asymmetric unit was composed of one formula unit, that is, six crystallographically inequivalent Ag^+^ ions, eight I^−^ ions together with one protonated [H_2_-4,4′-dpa]^2+^ cations (Figure 1a). Of note, all atoms in compound **1** are located in the 4e site with one site symmetry. In the [Ag_6_I_8_]*_n_*^2*n*−^ anionic moiety, all Ag centers adopted the tetrahedral geometries (Figure 1b), with the relevant Ag−I distances of 2.648(8)–3.126(5) Å. The angles of I−Ag−I spanned from 88.0(4) to 123.0(7)°, largely deviating from the ideal tetrahedral value of 109.5°. These observed results are reasonable and correlate well with some documented iodoargentates, such as [N-Bz-Py]_4_Ag_9_I_13_, [emIm]Ag_3_I_4_ (emIm = 1-ethyl-3-methyl imidazole), Hmta[(Hmta)Ag_4_I_4_] (Hmta = hexamethylenetetramine), [MCP]Ag_4_I_5_ (MCP^+^ = N-methyl-3-cyanopyridinium), [Co(bipy)_3_]Ag_3_I_6_ (bipy = 2,2′-bipyridine), [Cd(phen)_3_]_2_Ag_13_I_17_ (phen = 1,10-phenanthroline) and [Co(5,5′-dmpy)_3_]Ag_5_I_8_ (5,5′-dmbpy = 5,5′-dimethyl-2,2′-bipyridine) [3,5,8,12,17,20,21]. As for the I atoms, three different coordination modes exist: I(3) and I(6) atoms act as the bridging fashions; I(2), I(4), I(7) and I(8) atoms take on the μ_3_-I styles; while the I(1) and I(5) atoms are μ_4_-I manners connecting four Ag^+^ ions.

**Figure 2:**
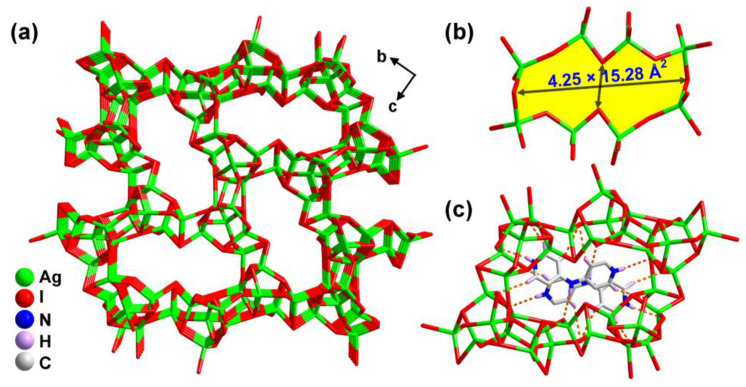
(**a**) Perspective view of 3D anionic framework of compound **1** along the *a* axis. (**b**) Detailed view of the peanut-typed window in compound **1**. (**c**) A diagram for showing the C−H···I hydrogen bonds between the [H_2_-4,4′-dpa]^2+^ cations and the [Ag_6_I_8_]*_n_*^2*n*−^ anions.

As depicted in Figure S1a, two [AgI_4_] tetrahedra shared one edge to produce two types of [Ag_2_I_4_] dimers, which were interfused by I(1) and I(4) atoms to obtain the tetranuclear [Ag_4_I_10_] subunit (Figure S1b). Then, the [Ag_4_I_10_] motif further joined two [AgI_4_] tetrahedra through sharing the I(1), I(3), I(5), I(6) and I(7) vertexs to generate the complicated [Ag_6_I_13_] moiety (Figure 1c), with the measured Ag···Ag separations in the range of 2.891(11)−3.299(2) Å. Such a value is evidently shorter than the sum of van der Waals radii of Ag (3.44 Å), implying the presence of argentophilic metal-metal interactions [22]. Every two neighboring [Ag_6_I_13_] moieties are held together by the I(2) atoms to form the 1D [Ag_6_I_12_]_n_^6n−^ chain (Figure 1d), which further linked the adjacent ones by the I(1), I(2), I(5) and I(8) atoms to promote the final 3D [Ag_6_I_8_]_n_^2n−^ anionic skeleton characteristic of the 1D channels (Figure 2a). The channels parallel to the a axis exhibited the peanut-shaped windows with a cross section of 4.25 × 15.28 Å^2^, which were defined by eight [AgI_4_] tetrahedra by corner-sharing (Figure 2b). The protonated [H_2_-4,4′-dpa]^2+^ cations as structure-affecting agents resided in the channels, resulting in the abundant hydrogen bonds with the I atoms (I(2), I(3), I(5), I(6) and I(7)) of the anionic network (Figure 2c). The C−H···I hydrogen lengths and angles are between 3.671(12)–3.991(13) Å and 129.0–170.6°, respectively (Table 1). As calculated by the PLATON program, the solvent-accessible volume excluding guest cations for compound **1** was found to be up to 33.3%. In addition, there also exist the versatile C−H···π and anion···π interactions in compound **1** (Figures S2 and S3).

Although compound [N-Bz-Py]_4_Ag_9_I_13_ and **1** are both 3D microporous materials decorated by similar template cations (Figure S4), their crystal structures emerge differently [5]. Concretely, the former features the [Ag_9_I_13_]*_n_*^4*n*−^ anionic fabrication containing [Ag_3_I_7_] and [Ag_6_I_12_] two types of SBUs, while compound **1** exhibits the [Ag_6_I_8_]*_n_*^2*n*−^ anion characteristic of unusual [Ag_6_I_13_] moieties. Another difference is reflected in their PBUs: Compound [N-Bz-Py]_4_Ag_9_I_13_ consists of [AgI_3_] triangles and [AgI_4_] tetrahedra two types of building blocks, while only the tetrahedral [AgI_4_] units appear in **1**. In addition, compared to some other microporous iodoargentates, the uniqueness of compound [H_2_-4,4′-dpa]Ag_6_I_8_ was still impressive. It is worth noting that there are also some analogs with the same Ag/I ratio as compound **1** isolated by the solvothermal method, such as [emIm]Ag_3_I_4_, [MCMP]Ag_3_I_4_, [EtPPh_3_]Ag_3_I_4_ (Et = ethyl; PPh_3_ = triphenylphosphine), [MPBI]Ag_3_I_4_ (MPBI^+^ = 1,3-dimethyl-2-phenylbenzimidazolium), [DMBTz]Ag_3_I_4_, [Hpy]_2_Ag_6_I_8_·DMF (Hpy^+^ = protonated pyridine; DMF = N,N′-dimethylformamide) and [V(DMSO)_5_(H_2_O)]Ag_6_I_8_ (DMSO = Dimethyl sulfoxide) [6,7,14,17,23,24,25]. Nevertheless, these documented examples normally crystallized in low-dimensional phrases, including the one-dimensional (1D) chains/ribbons and two-dimensional (2D) layers. For instance, compound [emIm]Ag_3_I_4_ possesses the layered [Ag_3_I_4_]*_n_^n^*^−^ architecture, while compound [DMBTz]Ag_3_I_4_ is the chain-like structure, which is constructed from the [Ag_3_I_8_] and [Ag_3_I_7_] SBUs, respectively [14,17]. More structural discrepancies between compound [H_2_-4,4′-dpa]Ag_6_I_8_ and some related iodoargentate derivatives are summarized in Table 2. Undoubtedly, compound **1** represents an unprecedented structural motif, making it unique in the numerous numbers of the iodoargentate family.

### 2.2. Hirshfeld Surface Analyses of Compound **1**

**Figure 3:**
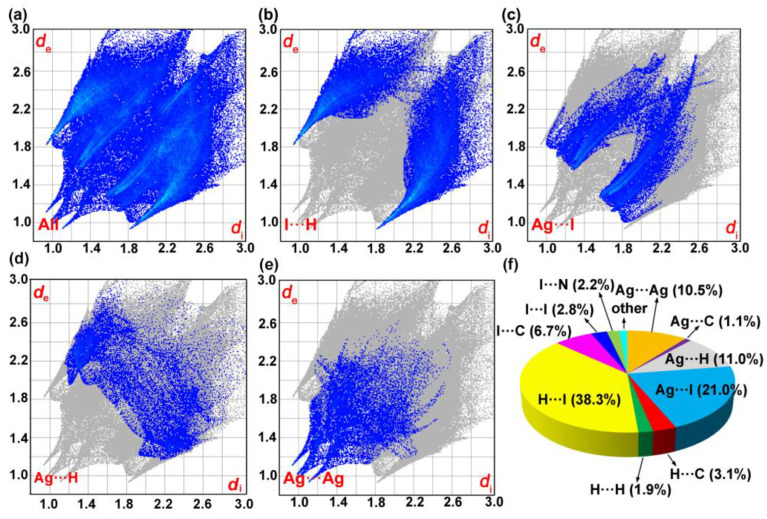
The 2D fingerprint plots of compound **1**: (**a**) Total interactions. (**b**) The H···I interactions. (**c**) The Ag···I interactions. (**d**) The Ag···H interactions. (**e**) The Ag···Ag interactions. (**f**) Contribution percentage of multiple interactions.

As a complementary to X-ray crystallography studies, we further performed the Hirshfeld surface analyses for compound [H_2_-4,4′-dpa]Ag_6_I_8_, which helped us to clearly recognize and quantitatively identify the noncovalent interactions across the molecular structure (Figure 3). The full 2D fingerprint plots, depicted in Figure 3a, covered the region of 2.0 ≤ d_e_ + d_i_ ≤ 6.0 Å, showing the contours bounding the effective electron density. Analyses of decomposed fingerprint plots revealed that the major contributor is due to the H···I interactions, with the sky-blue points scattering in the range of 2.8 ≤ d_e_ + d_i_ ≤ 5.4 Å (Figure 3b). They emerged like a pair of pseudo-symmetric wings, accounting for 38.3% of the total Hirshfeld surface (top left: 15.5%; bottom right: 22.8%). A high occupying ratio indicated that hydrogen bond interactions may play an important role in the stabilization of crystal packing. This has also appeared in the cases of some hydrogen-plentiful metal halides, such as [Co(5,5-dmpy)_3_]Ag_5_I_8_, [Zn(bipy)_3_]_2_Ag_2_BiI_6_(I)_1_._355_(I_3_)_1_._645_, [Fe(bipy)_3_]AgBiI_6_, [Co(bipy)_3_]_2_Ag_4_Bi_2_I_16_ and [(Me)_2_-(Dabco)]_2_Cu_2_Bi_2_I_12_ [21,27,28,29,30]. For compound **1**, the second contributor is derived from the Ag···I, which is represented by a lean tunnel and comprises 21.0% of full weak interactions (Figure 3c). In addition, the Ag···H and Ag···Ag contracts, are also non-negligible, with the proportions of 11.0% and 10.5%, respectively (Figure 3d,e). The remaining interatomic contacts, such as I···C, H···H and H···C, are provided in Figure S5. For example, the percentages of I···C and H···C contacts are found to be 6.7% and 3.1%, thereby confirming the presence of anion···π and C−H···π interactions. These results are well consistent with the analyses of single crystal X-ray diffraction. Comparison of multiple intra/inter-molecular interactions is displayed as a pie chart (Figure 3f).

### 2.3. Characterizations and Optical Behaviors of Compound **1**

**Figure 4:**
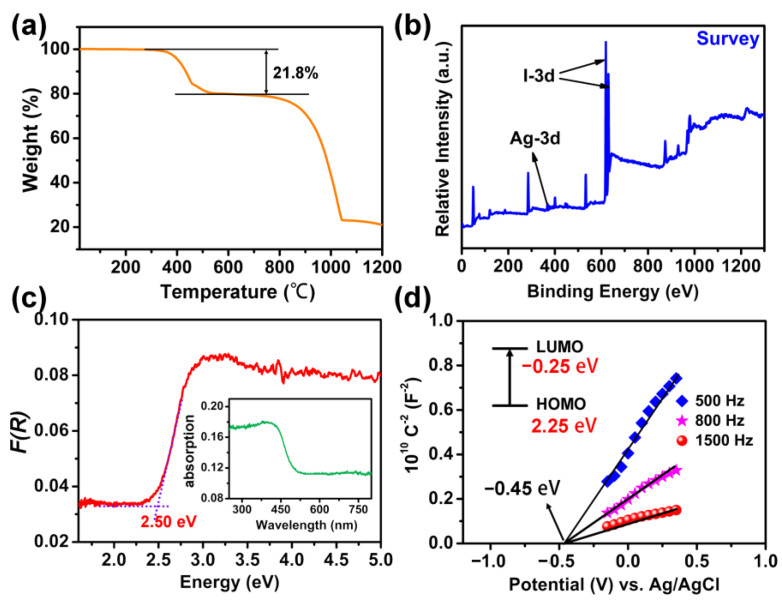
(**a**) Thermogravimetric curve of compound **1**. (**b**) XPS survey spectrum of compound **1**. (**c**) UV-Vis diffuse reflectance spectrum of compound **1**. Inset: The absorption spectrum. (**d**) Mott-Schottky plots of compound **1**. Inset: Energy diagram of the HOMO and LUMO levels.

As displayed in Figure S7, the experimental powder X-ray diffraction (PXRD) pattern matched well with the simulated result derived from single-crystal X-ray diffraction data, indicating the high phase purity of our as-grown crystals. Under the nitrogen environment, we next checked the thermal behavior of compound [H_2_-4,4′-dpa]Ag_6_I_8_, which was depicted in Figure 4a. It can be conspicuously seen that compound **1** experienced a two-step weight loss in the range of 30–1200 °C, with the preliminary weight change occurring at about 380 °C. Remarkably, this is in stark contrast with the poor thermal stability demonstrated by most metal halide hybrids, such as [NH_4_]_2_AgI_3_, [Zn(phen)_3_]_2_Ag_8_I_12_·7DMF, [Al(DMSO)_6_]Ag_9_I_12_, [HCP]Ag_2_I_3_ (HCP^+^ = NH-4-cyanopyridinium), [H_2_-Dabco][Ag_2_I_4_(Dabco)], [Et-btz]AgI_2_ (btz = benzothiazole) and [(Me)_2_-(Dabco)]_2_Cu_2_Bi_2_I_12_, whose decomposition temperatures were routinely as low as 200 °C [4,13,15,20,30,31,32]. The chemical compositions of compound [H_2_-4,4′-dpa]Ag_6_I_8_, i.e., C, N, Ag and I elements, were further confirmed by the EDX and XPS results (Figure S10 and Figure 4b). Analyses of the high-resolution Ag−3d spectrum showed that it contained two single peaks centered at binding energies of 373.9 and 367.9 eV, corresponding to the 3d_3/2_ and 3d_5/2_ states of Ag^+^ ions (Figure S11). The high-resolution I−3d spectrum was also characterized by two single peaks, with the binding energies located at 630.1 (3d_3/2_) and 618.6 (3d_5/2_) eV, respectively (Figure S12). These observed values are reasonable and are very close to some reported results (e.g., [(Me)_2_-2,2′-bipy]Ag_8_I_10_, [MNH]Ag_3_I_5_ ([MNH]^2+^ = methylated nicotinohydrazide) and [AE2T]_2_AgBiI_8_ (AE2T = 5,5′-diylbis(aminoethyl)-[2,2′-bithiophene]) [18,33,34].

To appreciate the semiconductor behavior of compound [H_2_-4,4′-dpa]Ag_6_I_8_, we then measured its UV-Vis diffuse reflectance and absorption spectra using a powdered sample at room temperature. As presented in the inset of Figure 4c, compound **1** has an absorption at 400–600 nm, with a steep absorption edge at about 496 nm. According to the Kubelka−Munk function, the optical band gap of compound **1** was estimated to be 2.50 eV (Figure 4c), suggesting its semiconducting nature. This energy gap was well comparable to the values of [V(DMSO)_5_(H_2_O)]Ag_6_I_8_ (2.61 eV), [MCP][Ag_4_I_5_] (2.63 eV), [Ag_2_I_2_(phen)] (2.45 eV), [AgI(bpt)] (bpt = 3,5-bis(pyrazinyl)-1,2,4-triazole; 2.53 eV), [Co(phen)_3_]Ag_2_I_4_·3DMF (2.59 eV) and [Co(phen)_3_]Ag_3_I_5_·DMF (2.58 eV) [13,20,25,35]. In addition, the band gap of compound [H_2_-4,4′-dpa]Ag_6_I_8_ exhibited a noticeable red shift with respect to the bulk β-AgI (2.81 eV), which was observed in the majority of iodoargentate hybrids, including but not limited to [(Me)_2_-2,2′-bipy]Ag_8_I_10_, [(Me)_2_-2,2′-bipy]_2_Ag_7_I_11_, [H_2_-bip]Ag_2_I_3_(µ-CHO) (bip = 2,6-bis(1-imdazoly)pyridine), AgI(bpt), [Co(bipy)_3_]Ag_3_I_6_ and K[Fe(bipy)_3_]_2_Ag_6_I_11_ [12,16,18,35,36,37].

Mott-Schottky plots depicted in Figure 4d were tested to further know the optical behavior of compound [H_2_-4,4′-dpa]Ag_6_I_8_. Evidently, the positive slope indicated the n-type semiconducting character of compound **1**, with the flat-band position of −0.45 eV versus Ag/AgCl. As is known to all, for n-type semiconductors, the flat band potential is customarily 0.20 V lower than the conduction band. Therefore, the conduction band of compound [H_2_-4,4′-dpa]Ag_6_I_8_ was determined to be −0.25 V vs. normal hydrogen electrode (NHE). Correspondingly, the valence band was evaluated as approximately 2.25 eV vs. NHE.

### 2.4. Photoelectric Performances of Compound **1**

**Figure 5:**
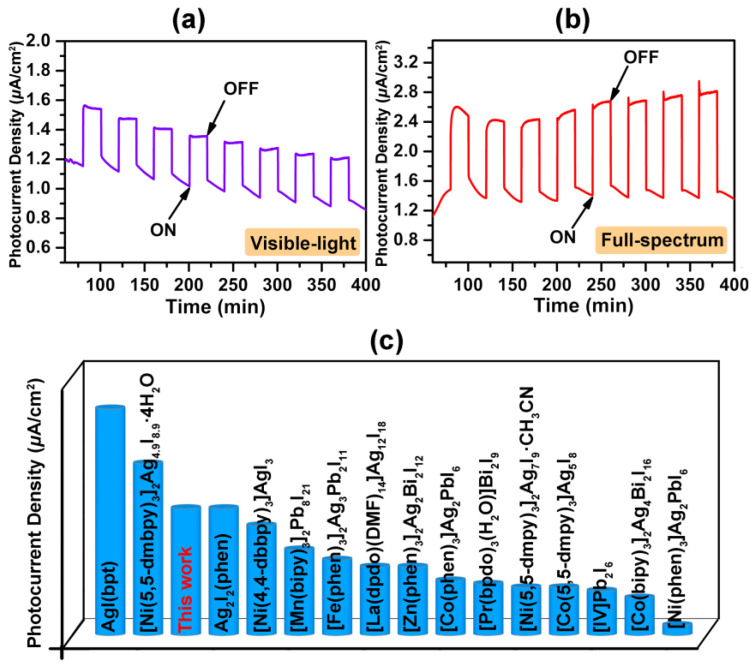
The photocurrent curves of compound **1**: Visible light (**a**) and full-spectrum (**b**). (**c**) The comparisons of photocurrent density between title compound and some representative analogues in literature.

Considering that the photoinduced current generation can be potentially applied in the fields of intelligent switch, information storage and communication transmission, we further executed the photoelectrochemical measurement for compound [H_2_-4,4′-dpa]Ag_6_I_8_. The photocurrent-time curves with the on/off status were recorded in Figure 5a,b. The rapid circuit photocurrent responses with negligible decays could be monitored under the periodic irradiation achieved by a manual shutter. Despite the illumination after several cycles, the photoelectric switching performance could be well preserved, indicating good moisture stability and performance durability. This is markedly different from some iodoplumbate-based hybrid materials (e.g., [CH_3_NH_3_]PbI_3_), which usually performed poorly due to the long-term instabilities [38]. Under the visible light condition, the average photocurrent density reached to 0.37 μA·cm^−2^ (Figure 5a), which is comparable to those of some highly reactive iodoargentate-based hybrids, such as AgI(bpt), Ag_2_I_2_(phen), [Co(5,5-dmpy)_3_]Ag_5_I_8_, [La(dpdo)(DMF)_14_]Ag_12_I_18_ (dpdo = 4,4′-bipyridine N,N’-dioxide) and [Co(bipy)_3_]_2_Ag_4_Bi_2_I_16_ [21,29,35,39]. Moreover, the on/off current density of compound [H_2_-4,4′-dpa]Ag_6_I_8_ could be further enhanced by exposing the designed photoelectrode to the full-spectrum circumstance, with the observed photocurrent density of 1.23 μA·cm^−2^ (Figure 5b). Such an enhancement can be attributed to the increased amount of photoexcited electrons and holes owing to the expanded spectral range, which has also appeared in some previous studies [27,28,29]. These results mean that compound **1** may serve as a promising light-harvesting and light-detecting candidate. In addition, the comparisons of photocurrent density between the title compound and some representative analogs in literature are illustrated in Figure 5c.

### 2.5. Theoretical Studies of Compound **1**

**Figure 6:**
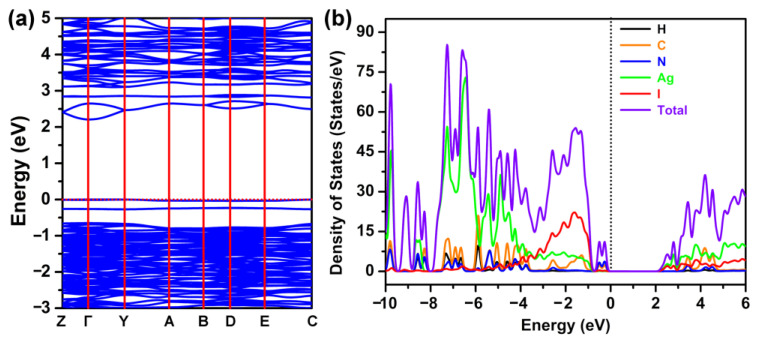
(**a**) The band structure of compound **1**. (**b**) DOS and partial DOS of compound **1**.

To gain a deeper correlation of the structure-property relationships, we performed the density functional theory (DFT) calculations for compound [H_2_-4,4′-dpa]Ag_6_I_8_ with the assistance of a first-principle approach. The electronic band structure, energy gap and density of states (DOS) were provided. As shown in Figure 6a, the band gap was found to be 2.23 eV under the GGA + U method, which agreed well with the experimental value of 2.50 eV. Noteworthily, this obviously distinguished the results obtained through conventional GGA, which generally significantly underestimated the true value. Analyses of band structures showed that the valence bands (VBs) maximum and conduction bands (CBs) minimum were both located at Γ points. Hence, the title compound can be considered as the quasi-direct bandgap semiconductor. In addition, the dispersive band seemed like a pocket at the CBs minimum, which may largely be benefiting the transport of photo-generated electrons.

The total DOS of compound [H_2_-4,4′-dpa]Ag_6_I_8_, as well as the partial DOS of C, N, H, Ag and I atoms, were given in Figure 6b and Figure S15. It can be seen that the VBs maximum predominantly corresponds to the 2p levels of N and C atoms, suggesting the obvious covalent interactions of C−N bonds. Furthermore, the contributions of N and C induced a few isolated bands of the upper VBs near 0 eV. The broad intensity peak near the top of the VBs was mainly dominated by I−5p and Ag−4d states, which revealed the presence of strong interactions between Ag and I atoms. The deeper VBs, for example, with energy less than −4 eV, were mainly contributed by Ag−4d orbits. Taking into account the strong localization of the d states of Ag, the flat valence band structures were further verified. Whereas the 4s states of Ag atoms and 5p states of I atoms dominated the DOS around the bottom of CBs. The s-p hybridization may be responsible for the CB dispersion, which was found to promote the photo-excited carrier transfers. Thus, from the above-mentioned calculated results, we can conclude that the electrons in the title compound behaved with higher carrier mobility than holes.

## 3. Materials and Methods

### 3.1. Reagents

Silver iodide (AgI, Adamas), 4,4′-dipyridylamine (4,4′-dpa, Adamas), potassium iodide (KI, Greagent), hydriodic acid (HI, Adamas) and acetonitrile (CH_3_CN, Kermel). All reagents were of analytical grades and were purchased from commercial sources, which were directly used in the preparation process unless otherwise stated.

### 3.2. Instruments and Measurements

The purity identifications of the title compound were completed by the powder X-ray diffractometer (Bruker D8, CuK*α* radiation) and elemental analyzer (German Elementar Vario EL Cube apparatus). The thermogravimetric behavior was evaluated by a NETZSCH STA449F3 unit with a heat rate of 10 K/min (N_2_ atmosphere). The UV-Vis absorption and diffuse reflectance patterns were monitored by a SHIMADZU UV-3600 spectrometer. Energy-dispersive X-ray spectrum studies were performed on a Thermo Fisher GX4 scanning electron microscope. A Thermo Scientific ESCLAB 250Xi spectrometer was used to acquire X-ray photoelectron spectroscopy diagrams.

### 3.3. Preparation of Compound **1**

117 mg of AgI (0.5 mmol), 166 mg of KI (1.0 mmol), and 34 mg of 4,4′-dpa (0.2 mmol) were put in 0.5 mL HI, 2 mL H_2_O and 4 mL CH_3_CN. Afterward, the resulting solution was sealed in a 23 mL polytetrafluoroethylene-lined container and kept heating for 5 days at 140 °C. Through the filtration and the ethanol washing, yellow sheet-type crystals were harvested by manual separation, with a yield of around 21% based on AgI. It is emphasized that the mixed HI/H_2_O/CH_3_CN solvent may have a significant impact on the crystallization of the title compound. Our extensive syntheses studies have shown that other reaction medium (such as methanol, ethanol, acetone, DMF and DMSO) was found to be unfavorable, often leading to a failure to obtain the targeted product. Elemental analysis (EA) calculated for compound **1**: C, 6.54%; H, 0.60%; N, 2.29%. Found: C, 6.72%; H, 0.65%; N, 2.30%.

### 3.4. X-ray Crystallography

Single-crystal X-ray diffraction data collection of **1** was accomplished on an Xcalibur E Oxford diffractometer using Mo-K*α* radiation (*λ* = 0.71073 Å) at 298(2) K. Its structure obtained by direct methods was then refined by the SHELXL-2018 program based on full matrix least-squares routines against *F*^2^ [40]. During the refinement, all non-hydrogen atoms were treated anisotropically, while the hydrogen atoms were positioned geometrically with fixed thermal factors. In compound **1**, the Ag atoms except Ag(6) behave the disorder: Ag(1)/Ag(1B), Ag(3)/Ag(3B) and Ag(5)/Ag(5B) exist two statistical distributions; while the Ag(2)/Ag(2B)/Ag(2C) and Ag(4)/Ag(4B)/Ag(4C) are three statistical distributions. The empirical formula was further verified by the element analyses and thermogravimetric results. More structural refinement parameters are listed in Table 3. CCDC number 2,301,354 corresponds to compound **1**, which was acquired free of charge from the Cambridge Crystallographic Data Centre. Some important bond distances and angles are supplied in Table S1.

### 3.5. Photoelectric Examinations

Utilizing a CHI660E electrochemical workstation (Chenhua, Shanghai, China) equipped with the three-electrode configuration, we examined the photoelectric switching performance of the title compound. The working electrode was prepared by a typical solution coating method that stated as follows: 5 mg of microcrystalline powder **1** was added into a mixed Nafion/ethanol solution, suffering from the ultrasonic treatment. After lasting for 30 min, the gained suspension was deposited on the clean surface of ITO glass and then dried in the air. The effective area was 1.0 × 1.0 cm^2^. In this study, Ag/AgCl and platinum wire served as the reference electrode and the counter electrode, respectively. A 300 W Xenon lamp was used as the irradiation source, while the visible light was realized with the help of a 420 nm cut-off filter. The supporting electrolyte is the 0.1 M KCl solution.

### 3.6. Computational Details

The electronic structure calculations of compound **1** were conducted by the density functional theory (DFT) framework implemented in the Vienna Ab initio Simulation Package (VASP) [41]. The Perdew–Burke–Ernzerhof (PBE) form of generalized gradient approximation (GGA) exchange-correlation functionals have been employed, utilizing projector augmented wave (PAW) potentials [42,43]. The H−1s, C−2s, C−2p, N−2s, N−2p, Ag−4d, Ag−5s, I−5s and I−5p were treated as its valence states. In order to escape the well-known bandgap underestimate of GGA, the Coulomb self-interaction potential was considered. Within the GGA + U approximation, the onsite Coulomb term U value was used for the Ag−4d states. The energy cut-off for the plane wave basis set was kept at 500 eV. The reciprocal space sampling was completed with *k*-point Monckhorst–Pack grids of 5 × 5 × 3 for the title compound.

## 4. Conclusions

In summary, using the solvothermal method, we successfully fabricated a new iodoargentate microporous material, which was subsequently structurally analyzed and characterized by means of multiple techniques. The title compound featured a novel and complicated 3D [Ag_6_I_8_]*_n_*^2*n*−^ anionic framework based on hexameric [Ag_6_I_13_] SBUs, containing the peanut-shaped 1D channels where the [H_2_-4,4′-dpa]^2+^ template cations reside. Further research showed that the obtained material exhibited semiconductive behavior, rendering it with good photoelectric conversion properties upon alternate light illumination. Of note, its photocurrent density competed well with or even surpassed those of some high-performance halide analogs, which was mainly attributed to the high mobility of electrons as revealed by theoretical calculations. Future work will focus on the exploratory syntheses of more new numbers and the deep investigation of their structure-property relationships.

## Figures and Tables

**Table 1:** Hydrogen bonds (Å) and angles (°) for compound **1**.

C−H···I	*d*(C−H)	*d*(H···I)	*d*(C···I)	<(CHI)
N(1)−H(1)···I(5)#9	0.86	2.92	3.691(12)	150.5
N(2)−H(2A)···I(3)#4	0.86	2.87	3.718(10)	170.6
N(3)−H(3)···I(3)#8	0.86	3.05	3.892(12)	165.7
C(1)−H(1A)···I(6)#9	0.93	3.05	3.903(12)	153.9
C(4)−H(4)···I(6)#8	0.93	3.30	3.991(13)	133.2
C(8)−H(8)···I(7)#2	0.93	3.03	3.930(17)	164.7
C(9)−H(9)···I(2)#8	0.93	3.07	3.873(16)	145.5
C(10)−H(10)···I(6)#8	0.93	3.27	3.920(15)	129.0

Symmetry transformations used to generate equivalent atoms: #2 *x* + 1, *y*, *z*; #4 −*x* + 1, −*y* + 1, −*z*; #8 −*x* + 3/2, *y* + 1/2, −*z* + 1/2; #9 −*x* + 1, −*y* + 2, −*z*.

**Table 2:** The structural comparisons of compound **1** with some related iodoargentate derivatives.

Compound	D	Template	SG	PBUs	SBUs	I	Reference
[H_2_-4,4′-dpa]Ag_6_I_8_	3	[H_2_-4,4′-dpa]^2+^	P2_1_/n	[AgI_4_]	[Ag_6_I_13_]	μ-2; μ-3; μ-4	This work
[*N*-Bz-Py]_4_Ag_9_I_13_	3	[*N*-Bz-Py]^+^	Cc	[AgI_3_], [AgI_4_]	[Ag_3_I_7_], [Ag_6_I_12_]	μ-2; μ-3; μ-4	[5]
[(Me)_2_-2,2′-bipy]Ag_8_I_10_	3	[(Me)_2_-2,2′-bipy]^2+^	C2/c	[AgI_4_]	[Ag_8_I_15_]	μ-3; μ-4	[18]
[Mg(en)_3_]Ag_2_I_4_	3	[Mg(en)_3_]^2+^	P6_3_22	[AgI_4_]	[AgI_4_]	μ-2	[9]
[Co(phen)_3_]_2_Ag_13_I_17_	3	[Co(phen)_3_]^2+^	P2_1_3	[AgI_4_]	[Ag_6_I_13_], [Ag_7_I_13_]	μ-2; μ-3; μ-4	[3]
[DMBTz]_2_Ag_5_I_7_	3	[DMBTz]^+^	C2/c	[AgI_4_]	[Ag_5_I_9_]	μ-1; μ-2; μ-3; μ-4	[19]
[H_3_(Dabco)_2_]Ag_3_I_6_	3	[H_3_(Dabco)_2_]^3+^	R32	[AgI_4_]	[Ag_3_I_9_]	μ-2	[26]
Hmta[(Hmta)Ag_4_I_4_]	3	Hmta	F-43m	[AgI_3_]	[Ag_4_I_4_]	μ-3	[8]
[(Hmta)_2_Ag_8_I_6_]I_2_	3	Hmta	Fm-3m	[AgI_3_]	[Ag_8_I_6_]	μ-4	[8]
[emIm]Ag_3_I_4_	2	[emIm]^+^	Pccn	[AgI_4_]	[Ag_3_I_8_]	μ-2; μ-3	[17]
[MCMP]Ag_3_I_4_	3	[MCMP]^+^	C2/c	[AgI_4_]	[Ag_6_I_12_]	μ-2; μ-3; μ-4	[6]
[EtPPh_3_]Ag_3_I_4_	1	[EtPPh_3_]^+^	P2_1_/c	[AgI_4_]	[Ag_3_I_7_]	μ-2; μ-3; μ-4	[23]
[MPBI]Ag_3_I_4_	2	[MPBI]^+^	Pnna	[AgI_4_]	[Ag_3_I_8_]	μ-3	[24]
[DMBTz]Ag_3_I_4_	1	[DMBTz]^+^	P2_1_/c	[AgI_4_]	[Ag_3_I_7_]	μ-2; μ-3; μ-4	[14]
[Hpy]_2_Ag_6_I_8_·DMF	2	[Hpy]^+^	Pccn	[AgI_4_]	[Ag_3_I_8_]	μ-3	[7]
[V(DMSO)_5_(H_2_O)]Ag_6_I_8_	2	[V(DMSO)_5_]^2+^	P2_1_/c	[AgI_4_]	[Ag_5_I_10_]	μ-2; μ-3; μ-4	[25]

D = dimension; SG = Space group; en = ethylenediamine; Dabco = 1,4-diazabicyclo [2.2.2]octane.

**Table 3:** Crystallographic data and structural refinement details of compound **1**.

	Compound 1
CCDC	2,301,354
formula	C_10_H_11_Ag_6_I_8_N_3_
weight	1835.64
temperature/K	298(2)
wavelength/Å	0.71073
crystal system	monoclinic
space group	*P*2_1_/*n*
*a/*Å	11.3797(11)
*b/*Å	14.1621(13)
*c/*Å	18.8035(17)
*β* */* *°*	104.387(4)
volume/Å^3^	2935.3(5)
Z	4
*D*_calcd_/g·cm^−3^	4.154
*μ*/mm^−1^	12.343
*F*(000)	3192
reflection collected	13,260
unique reflection	5184
*R*_int_	0.0249
*R*_1_ [*I* > 2*s*(*I*)], *wR*_2_ [*I* > 2*s*(*I*)]	0.0415, 0.0955
*R*_1_ [all data], *wR*_2_ [all data]	0.0551, 0.1030

## Data Availability

Data are contained within the article and supplementary materials.

## Supplementary Materials

The following supporting information can be downloaded at: https://www.mdpi.com/article/10.3390/molecules28248033/s1, Table S1: Selected bond lengths (Å) and bond angles (°) for compound **1**. Figure S1: (a) Two types of [Ag_2_I_6_] dimers. (b) The [Ag_4_I_10_] unit. (c) The [Ag_6_I_13_] moiety formed by [Ag_4_I_10_] unit and two [AgI_4_] tetrahedra; Figure S2: A pair of [H_2_-4,4′-dpa]^2+^ cations showing the C−H···*π* interactions; Figure S3: The anion···*π* interactions existing in compound **1**; Figure S4: (a) The 4,4′-dpa ligand in compound **1**. (b) The *N*-Bz-Py ligand in [*N*-Bz-Py]_4_Ag_9_I_13_; Figure S5: Fingerprint plots: resolved into I···C (a), H···C (b), I···I (c), I···N (d), H···H (e) and Ag···C (f) for compound **1**; Figure S6: Hirshfeld surfaces analyses mapped with *d*_norm_ (a), *d*_i_ (b), *d*_e_ (c) and curvedness (d) for compound **1**; Figure S7: Experimental and simulated PXRD patterns of compound **1**; Figure S8: PXRD of pristine sample and the sample immersed in aqueous solution for one day; Figure S9: PXRD of pristine sample and the sample after photocurrent measurement; Figure S10: EDX spectrum of compound **1**; Figure S11: High-resolution Ag−3d peaks of compound **1**; Figure S12: High-resolution I−3d peaks of compound **1**; Figure S13: (a) Photocurrent-time curves of 4,4′-dpa ligand. (b) Photocurrent-time curves of blank ITO; Figure S14: Illumination lifetime of compound **1**; Figure S15: Total density of states and partial density of states for compound **1**. The Fermi level is set at 0 eV (dotted line).
