# Supplementary material for: A New 3D Iodoargentate Hybrid: Structure, Optical/Photoelectric Performance and Theoretical Research
Source: Molecules. 2023 Dec 10;28(24):8033. doi: 10.3390/molecules28248033 (PMC10745466; doi:10.3390/molecules28248033)
Supplement: Supplementary file 1 [file molecules-28-08033-s001.zip › molecules-2737029-supplementary.pdf]

Supporting Information

A New 3D Iodoargentate Hybrid: Structure, Optical/Photoelectric Performance and Theoretical Research

Jun Li [1,2*], Shuyue Xie [1], Ming Pang [1], Jiacheng Zhu [1], Jinting Wu [1], Yongdi Zhang [1], and Bo Zhang [1,2*]

[1]*College of Chemistry and Chemical Engineering, Shandong Provincial Key Laboratory and Collaborative Innovation Center of Chemical Energy Storage & Novel Cell Technology, Liaocheng University, Liaocheng, 252059, China*

[2]*State Key Laboratory of Structural Chemistry, Fujian Institute of Research on the Structure of Matter, Chinese Academy of Sciences, Fuzhou, 350002, China*

[*]*Correspondence: junli@lcu.edu.cn; bzhang@lcu.edu.cn*

Table of Contents

Table S1: Additional structural details.

Figures S1−S4: Crystal structures.

Figures S5−S6: Hirshfeld surface analyses.

Figures S7−S9: PXRD patterns.

Figure S10: EDX spectrum.

Figures S11−S12: XPS spectra.

Figure S13: Photocurrent-time curves.

Figure S14: Illumination lifetime.

Figure S15: DFT calculations.

Reference

1. Additional structural details

Table S1 Selected bond lengths (Å) and bond angles (°) for compound **1**.

Ag(1)−I(6)#1	2.806(2)	Ag(2B)−I(1)#3	2.764(5)
Ag(1)−I(7)#2	2.8505(19)	Ag(2B)−I(2)	2.807(5)
Ag(1)−I(1)	2.900(2)	Ag(2B)−I(5)#4	2.898(6)
Ag(1)−I(2)	2.900(3)	Ag(2B)−I(1)	2.923(6)
Ag(1)−Ag(2)	3.112(3)	Ag(3B)−I(2)#4	2.804(6)
Ag(2)−I(5)#2	2.844(2)	Ag(3B)−I(5)	2.850(6)
Ag(2)−I(1)#3	2.918(2)	Ag(3B)−I(1)#6	2.931(6)
Ag(2)−I(7)#2	2.925(2)	Ag(3B)−I(3)	2.932(6)
Ag(2)−I(1)	2.974(2)	Ag(4B)−I(3)	2.672(3)
Ag(2)−Ag(6)#2	3.299(2)	Ag(4B)−I(4)	2.769(3)
Ag(3)−I(3)	2.7625(17)	Ag(4B)−I(8)#5	2.876(3)
Ag(3)−I(2)#4	2.7893(16)	Ag(4B)−Ag(5B)#5	2.891(11)
Ag(3)−I(2)	2.8155(17)	Ag(4B)−Ag(6)#5	3.001(4)
Ag(3)−I(5)	2.9039(17)	Ag(4B)−I(7)#5	3.126(5)
Ag(3)−Ag(3)#4	3.031(3)	Ag(5B)−I(6)	2.648(8)
Ag(4)−I(3)	2.802(2)	Ag(5B)−I(4)#7	2.802(9)
Ag(4)−I(4)	2.876(2)	Ag(5B)−I(8)	2.810(9)
Ag(4)−I(8)#5	2.934(3)	Ag(2C)−I(2)	2.676(15)
Ag(4)−Ag(5)#5	3.007(4)	Ag(2C)−I(1)#3	2.789(15)
Ag(4)−I(1)#6	3.010(3)	Ag(2C)−I(1)	2.902(16)
Ag(5)−I(6)	2.778(5)	Ag(2C)−I(7)#2	3.075(17)
Ag(5)−I(8)	2.829(4)	Ag(4C)−I(1)#6	2.786(16)
Ag(5)−I(4)#7	2.889(4)	Ag(4C)−I(4)	2.83(2)
Ag(5)−I(5)	2.955(4)	Ag(4C)−I(3)	2.876(19)
Ag(5)−Ag(6)	3.102(6)	Ag(6)−I(7)	2.8000(14)
Ag(1B)−Ag(5B)#1	2.43(5)	Ag(6)−I(4)	2.8110(15)
Ag(1B)−I(6)#1	2.795(7)	Ag(6)−I(8)	2.8286(14)
Ag(1B)−I(1)	2.807(6)	Ag(6)−I(5)	2.9340(15)
Ag(1B)−I(7)#2	2.845(6)	Ag(2B)−I(1)#3	2.764(5)
Ag(2B)−Ag(3B)#4	1.974(8)		
I(6)#1−Ag(1)−I(7)#2	111.93(7)	I(2)−Ag(2B)−I(5)#4	97.47(15)
I(6)#1−Ag(1)−I(1)	114.91(8)	I(1)#3−Ag(2B)−I(1)	102.73(15)
I(7)#2−Ag(1)−I(1)	116.25(7)	I(2)−Ag(2B)−I(1)	113.62(19)
I(6)#1−Ag(1)−I(2)	110.97(8)	I(5)#4−Ag(2B)−I(1)	111.8(2)
I(7)#2−Ag(1)−I(2)	88.25(7)	I(2)#4−Ag(3B)−I(5)	98.65(18)
I(1)−Ag(1)−I(2)	111.56(8)	I(2)#4−Ag(3B)−I(1)#6	112.0(2)
I(5)#2−Ag(2)−I(1)#3	113.56(7)	I(5)−Ag(3B)−I(1)#6	110.93(19)
I(5)#2−Ag(2)−I(7)#2	105.69(6)	I(2)#4−Ag(3B)−I(3)	111.53(19)
I(1)#3−Ag(2)−I(7)#2	117.92(7)	I(5)−Ag(3B)−I(3)	107.42(19)
I(5)#2−Ag(2)−I(1)	109.88(7)	I(1)#6−Ag(3B)−I(3)	115.0(2)
I(1)#3−Ag(2)−I(1)	97.91(6)	I(3)−Ag(4B)−I(4)	115.26(12)
I(7)#2−Ag(2)−I(1)	111.75(7)	I(3)−Ag(4B)−I(8)#5	120.82(12)
I(3)−Ag(3)−I(2)#4	117.37(6)	I(4)−Ag(4B)−I(8)#5	116.97(10)
I(3)−Ag(3)−I(2)	114.83(6)	I(3)−Ag(4B)−I(7)#5	94.01(11)

I(2)#4−Ag(3)−I(2)	114.53(5)	I(4)−Ag(4B)−I(7)#5	94.58(11)
I(3)−Ag(3)−I(5)	110.67(5)	I(8)#5−Ag(4B)−I(7)#5	107.47(12)
I(2)#4−Ag(3)−I(5)	97.73(5)	I(6)−Ag(5B)−I(4)#7	119.5(4)
I(2)−Ag(3)−I(5)	98.23(5)	I(6)−Ag(5B)−I(8)	122.3(3)
I(3)−Ag(4)−I(4)	108.06(8)	I(4)#7−Ag(5B)−I(8)	118.1(3)
I(3)−Ag(4)−I(8)#5	114.53(10)	I(2)−Ag(2C)−I(1)#3	120.9(6)
I(4)−Ag(4)−I(8)#5	111.85(9)	I(2)−Ag(2C)−I(1)	118.6(7)
I(3)−Ag(4)−I(1)#6	116.58(9)	I(1)#3−Ag(2C)−I(1)	102.7(4)
I(4)−Ag(4)−I(1)#6	113.90(10)	I(2)−Ag(2C)−I(7)#2	88.0(4)
I(8)#5−Ag(4)−I(1)#6	91.30(6)	I(1)#3−Ag(2C)−I(7)#2	117.2(6)
I(6)−Ag(5)−I(8)	117.05(13)	I(1)−Ag(2C)−I(7)#2	109.5(5)
I(6)−Ag(5)−I(4)#7	112.37(19)	I(1)#6−Ag(4C)−I(4)	123.0(7)
I(8)−Ag(5)−I(4)#7	114.60(12)	I(1)#6−Ag(4C)−I(3)	121.7(7)
I(6)−Ag(5)−I(5)	107.94(10)	I(4)−Ag(4C)−I(3)	107.4(6)
I(8)−Ag(5)−I(5)	109.65(19)	I(7)−Ag(6)−I(4)	111.25(5)
I(4)#7−Ag(5)−I(5)	92.11(10)	I(7)−Ag(6)−I(8)	118.68(5)
I(6)#1−Ag(1B)−I(1)	118.3(3)	I(4)−Ag(6)−I(8)	104.22(5)
I(6)#1−Ag(1B)−I(7)#2	112.4(3)	I(7)−Ag(6)−I(5)	106.62(4)
I(1)−Ag(1B)−I(7)#2	119.5(2)	I(4)−Ag(6)−I(5)	105.04(5)
I(1)#3−Ag(2B)−I(2)	117.2(2)	I(8)−Ag(6)−I(5)	110.28(4)
I(1)#3−Ag(2B)−I(5)#4	114.52(19)		

Symmetry transformations used to generate equivalent atoms: #1 −*x*+3/2, *y*−1/2, −*z*+1/2; #2 *x*+1, *y*, *z*; #3 −*x*+2, −*y*+1, −*z*; #4 −*x*+1, −*y*+1, −*z*; #5 −*x*+1/2, *y*−1/2, −*z*+1/2; #6 *x*−1, *y*, *z*; #7 −*x*+1/2, *y*+1/2, −*z*+1/2; #8 −*x*+3/2, *y*+1/2, −*z*+1/2.

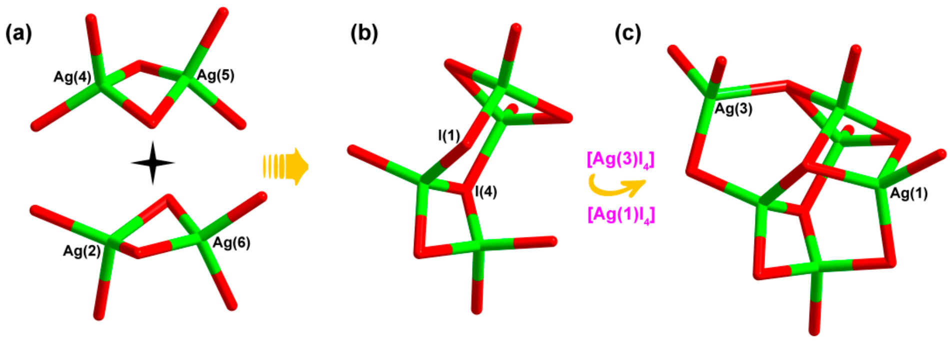

Figure S1 (a) Two types of $[Ag_2I_6]$ dimers. (b) The $[Ag_4I_{10}]$ unit. (c) The $[Ag_6I_{13}]$ moiety formed by $[Ag_4I_{10}]$ unit and two $[AgI_4]$ tetrahedra.

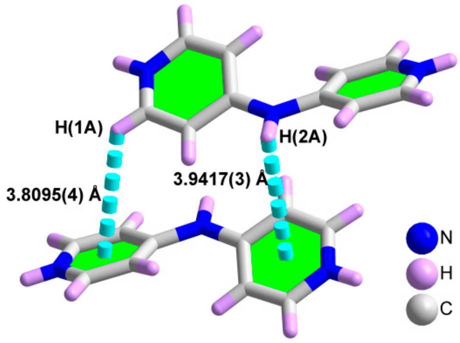

Figure S2 A pair of $[H_2\text{-}4,4'\text{-dpa}]^{2+}$ cations showing the C−H···π interactions.

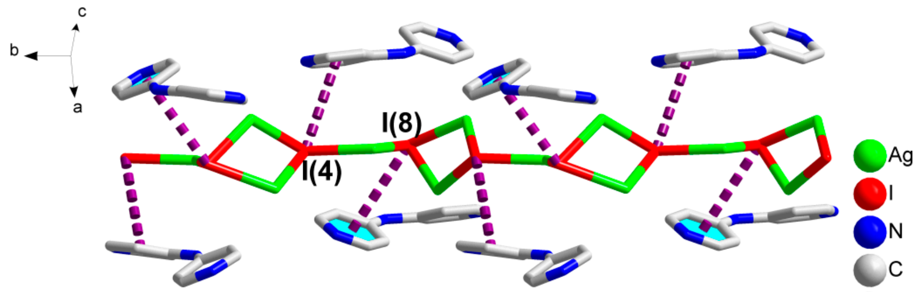

Figure S3 The anion···π interactions existing in compound **1**.

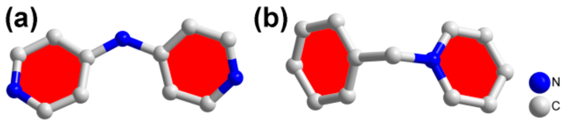

Figure S4 (a) The 4,4'-dpa ligand in compound **1**. (b) The *N*-Bz-Py ligand in [*N*-Bz-Py]$_4$Ag$_9$I$_{13}$[1].

2. Hirshfeld surface analyses

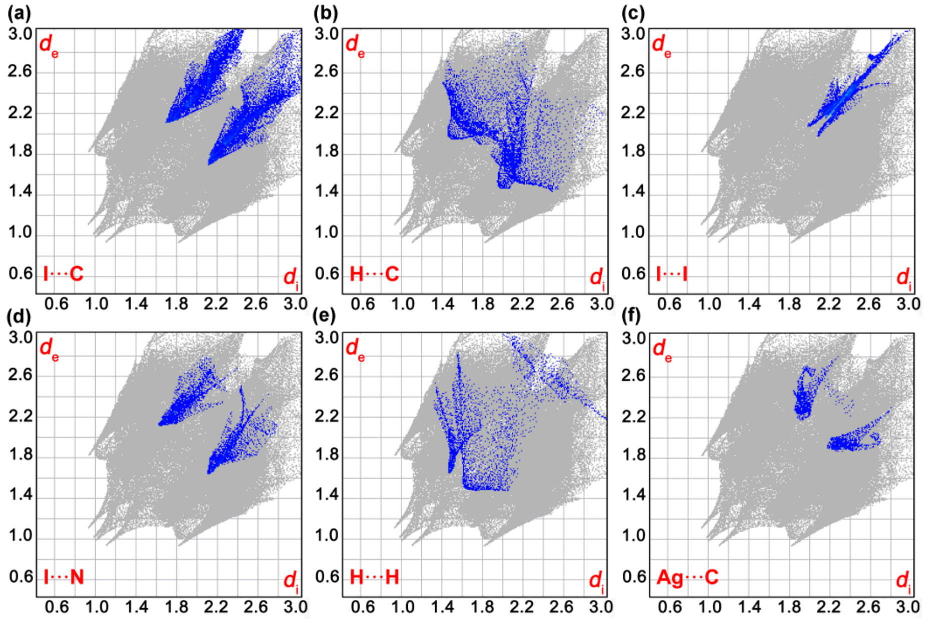

Figure S5 Fingerprint plots: resolved into I···C (a), H···C (b), I···I (c), I···N (d), H···H (e) and Ag···C (f) for compound **1**.

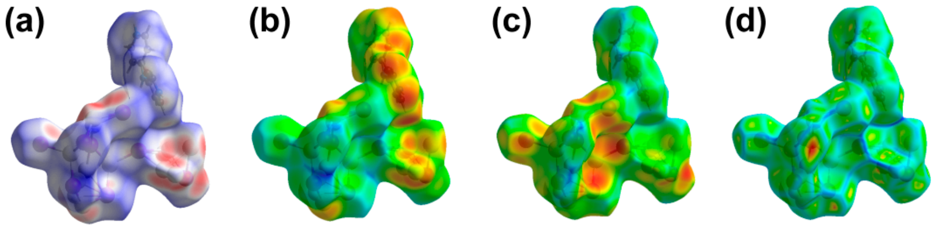

Figure S6 Hirshfeld surfaces analyses mapped with d_{norm} (a), d_i (b), d_e (c) and curvedness (d) for compound **1**.

3. Physical measurements

3a) PXRD

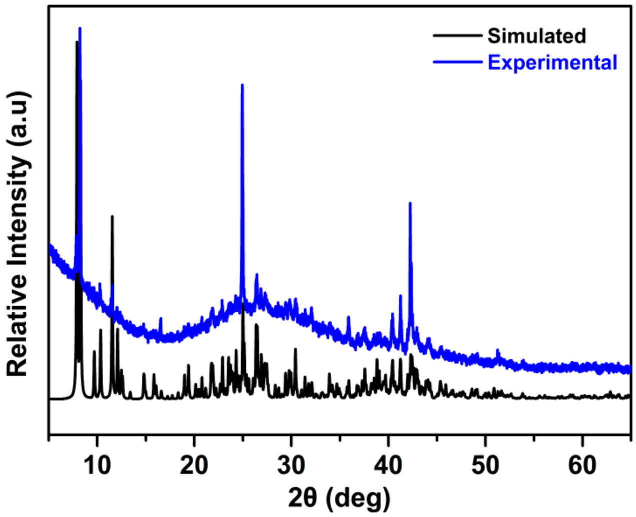

Figure S7 Experimental and simulated PXRD patterns of compound **1**.

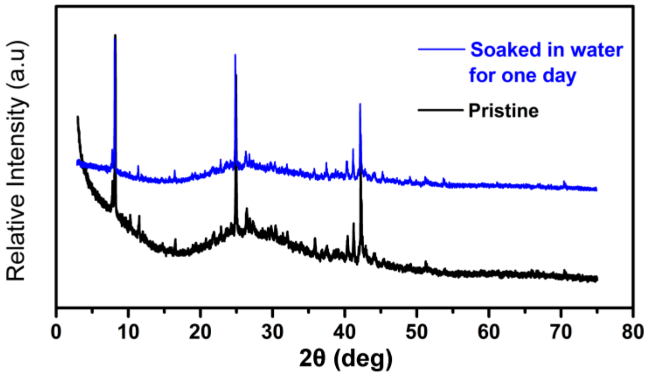

Figure S8 PXRD of pristine sample and the sample immersed in aqueous solution for one day.

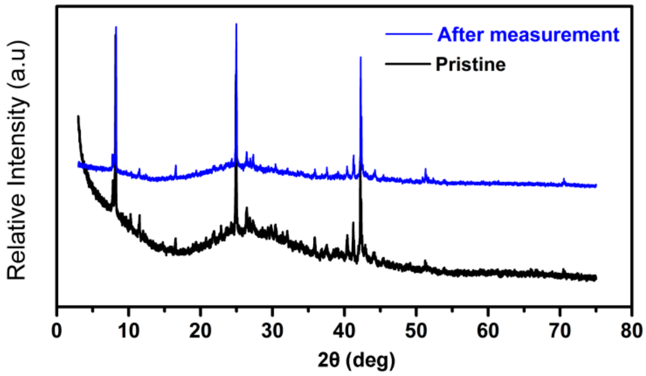

Figure S9 PXRD of pristine sample and the sample after photocurrent measurement.

3b) EDX

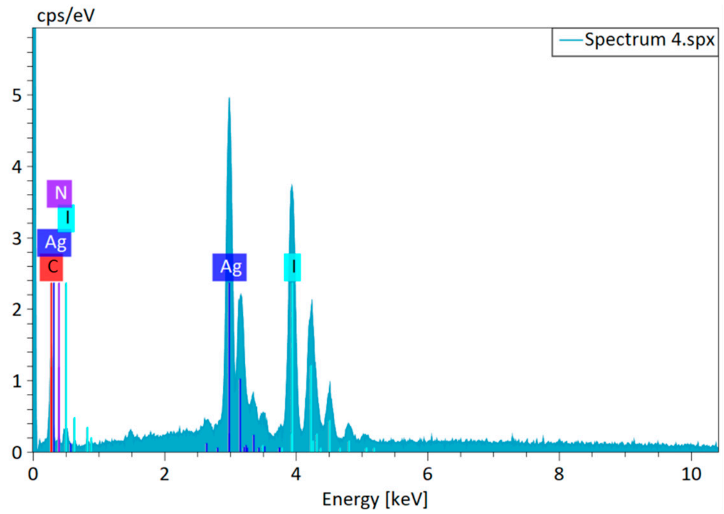

Figure S10 EDX spectrum of compound **1**.

3c) XPS

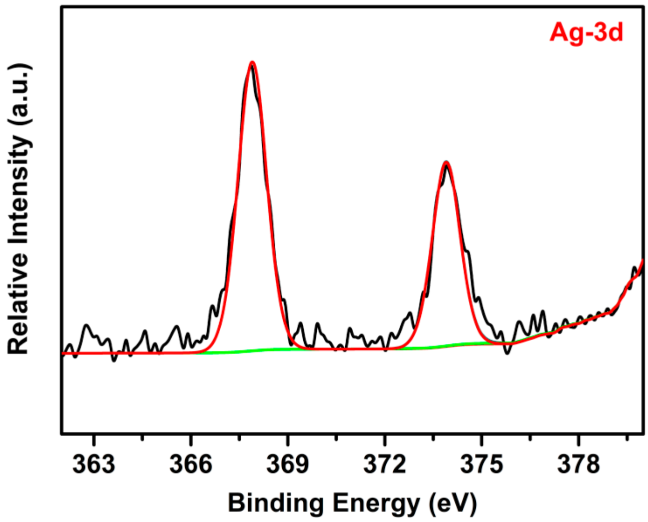

Figure S11 High-resolution Ag–3d peaks of compound **1**.

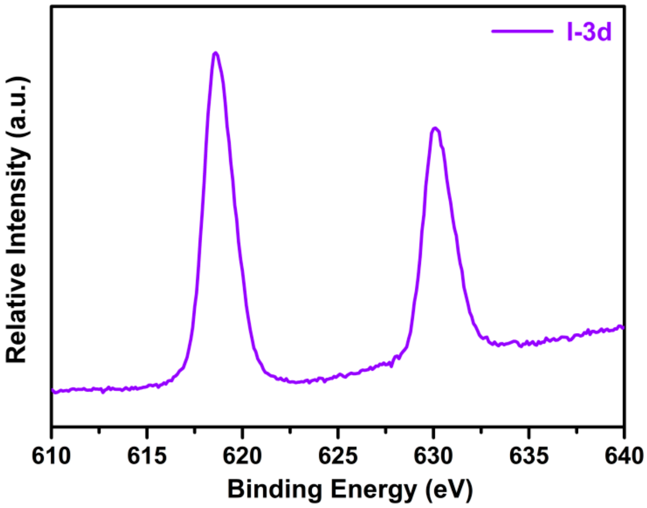

Figure S12 High-resolution I–3d peaks of compound **1**.

3d) Photocurrent

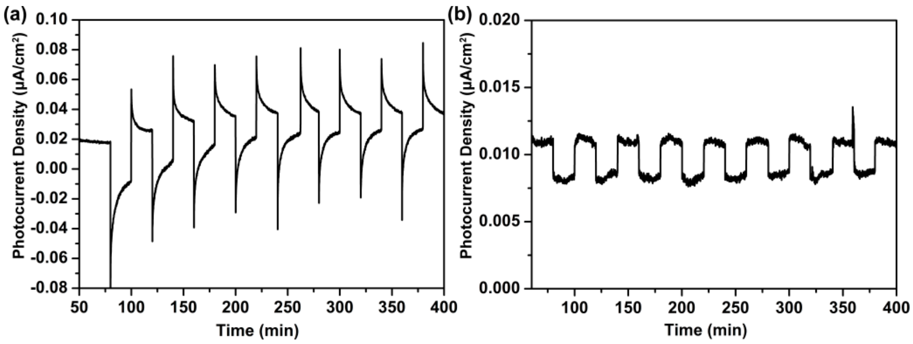

Figure S13 (a) Photocurrent-time curves of 4,4'-dpa ligand. (b) Photocurrent-time curves of blank ITO.

3e) Illumination lifetime

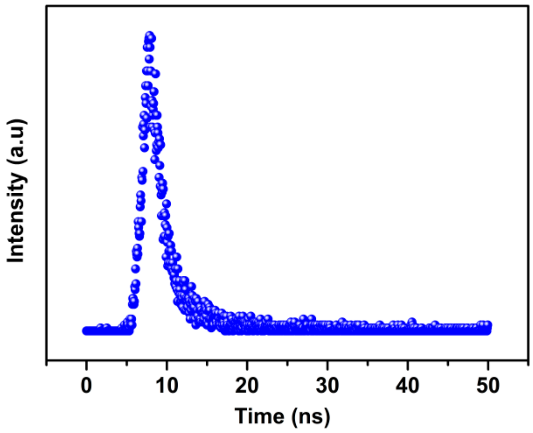

Figure S14 Illumination lifetime of compound **1**.

4. Theoretical calculations

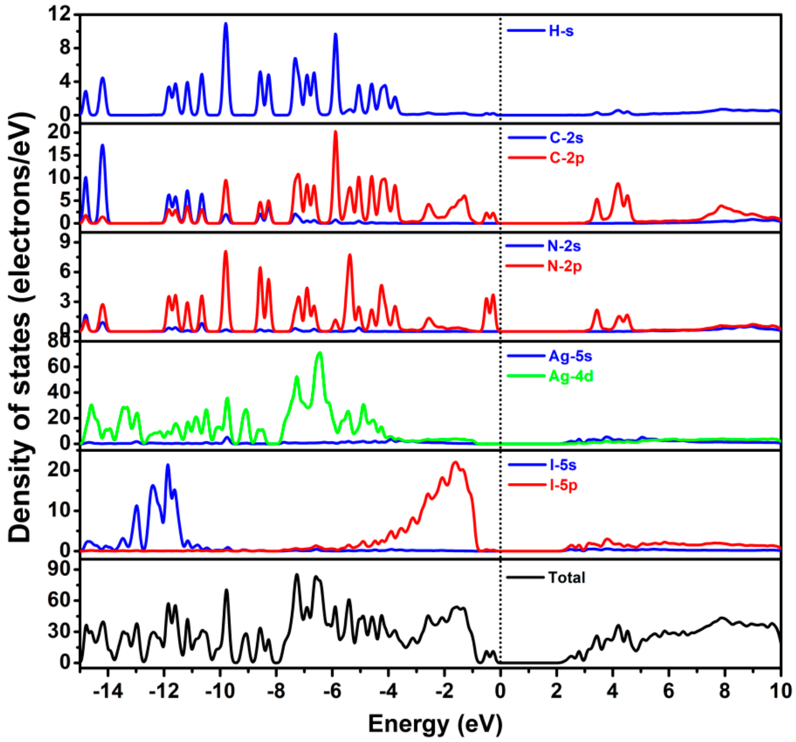

Figure S15 Total density of states and partial density of states for compound **1**. The Fermi level is set at 0 eV (dotted line).

5. Reference:

[1] Qiao, Y. R.; Hao, P. F; Fu, Y. L. Symmetrically related construction and optical properties of two noncentrosymmetric 3D iodides of d^{10} cation (Cu^+, Ag^+) based on the *N*-benzylpyridinium and its supramolecular interactions. *Inorg. Chem.* **2015**, *54*, 8705−8710.
